# Giant pulmonary hydatid cyst complicated by refractory shock and fulminant acute respiratory distress syndrome: a case report

**DOI:** 10.1097/RC9.0000000000000901

**Published:** 2026-08-25

**Authors:** Mohammad Alaa Aldakak, Mohammad Abdulghani, Raneem Ahmad, Youssef Abbas, Bassam Darwish

**Affiliations:** a Faculty of Medicine, Damascus University, Damascus, Syrian Arab Republic; b Department of General Surgery of the Faculty of Medicine, Damascus University, Damascus, Syrian Arab Republic

**Keywords:** acute respiratory distress syndrome, case report, hydatid cyst, pulmonary echinococcosis, re-expansion pulmonary edema

## Abstract

**Introduction:**

Giant pulmonary hydatid cysts are uncommon and may cause profound cardiopulmonary compromise through chronic lung compression and mediastinal displacement. Their surgical removal can be complicated by abrupt hemodynamic and respiratory deterioration.

**Case presentation:**

A 19-year-old woman from a rural livestock-raising region presented with progressive dyspnea, cough, and right-sided chest pain. Imaging demonstrated a solitary giant pulmonary cyst measuring approximately 40 × 25 × 25 cm, occupying almost the entire right hemithorax and markedly displacing the mediastinum. Serology for *Echinococcus granulosus* was positive. Through a right posterolateral thoracotomy, approximately 4 L of clear cyst fluid was aspirated, the endocyst was removed, bronchial openings were closed, and capitonnage was performed. Immediately after decompression, severe hypotension developed and was treated for suspected anaphylaxis with epinephrine and norepinephrine. Postoperatively, the patient remained intubated and developed early bilateral pulmonary infiltrates, severe hypoxemic respiratory failure, metabolic acidosis, and refractory shock. Echocardiography showed preserved biventricular function. Despite intensive care support, she died on the third postoperative day. The deterioration was considered multifactorial, with possible contributions from hypersensitivity, systemic inflammatory response, rapid decompression, re-expansion lung injury, and occult airway trauma.

**Conclusion:**

Giant pulmonary hydatid cysts should be regarded as high-risk physiological lesions even when surgical excision is technically successful. Controlled decompression, gradual lung re-expansion, multidisciplinary perioperative planning, and immediate access to advanced critical care are essential.

## Introduction

Cystic echinococcosis (CE) is a zoonotic infection caused by the larval stage of *Echinococcus granulosus* and is listed by the World Health Organization among the neglected tropical diseases. It is highly prevalent in livestock-raising areas, particularly across the Mediterranean basin, the Middle East, Central Asia, and South America. Community-based surveys from these regions report prevalence rates of approximately 5%–10%, while hospital series describe incidence rates reaching about 50 cases per 100 000 population, with pulmonary involvement representing around 20% of all cases^[^1,2^]^. Clinical manifestations largely reflect the site of involvement and the presence of complications. In pulmonary CE, small or early cysts are frequently asymptomatic; however, as cysts enlarge, rupture, or become secondarily infected, patients typically develop cough, chest pain, dyspnea, or hemoptysis. These features overlap considerably with those of pulmonary tuberculosis and lung abscess, which can lead to diagnostic confusion^[^3^]^. Diagnosis of pulmonary CE is based mainly on imaging. Chest radiography usually shows a well-defined cystic opacity, whereas computed tomography (CT) provides superior delineation of the cyst wall, internal membranes, and radiologic signs of rupture or superimposed infection. Serologic assays, including enzyme-linked immunosorbent assay and indirect hemagglutination, may support the diagnosis but generally have lower sensitivity for pulmonary hydatid cysts^[^4,5^]^. Therapeutic approaches are tailored to cyst size and complexity. Large, symptomatic, or complicated cysts typically warrant surgical management^[^6^]^. Because the lung is highly compliant, pulmonary hydatid cysts may attain considerable size, a phenomenon observed more frequently in children and young adults^[^7^]^. Here, we report an uncommon giant pulmonary hydatid cyst occupying almost the entire right hemithorax in a young woman, followed by refractory intraoperative shock and fatal postoperative acute respiratory distress syndrome despite technically successful surgical excision. This case highlights the unpredictable physiological hazards associated with decompression and re-expansion of a chronically compressed lung. The case has been reported in accordance with the SCARE 2025 guidelines^[^8^]^.

HIGHLIGHTS

- Giant pulmonary hydatid cysts may remain clinically occult until severe mass effect develops.
- Technically successful excision does not eliminate the risk of catastrophic physiological deterioration.
- Sudden shock after cyst decompression may reflect overlapping hypersensitivity, hemodynamic, and inflammatory mechanisms.
- Controlled decompression, gradual lung re-expansion, and multidisciplinary critical-care planning are essential.

## Case presentation

A 19-year-old woman, previously healthy and with no history of smoking or alcohol use, presented to our thoracic surgery department with progressive respiratory symptoms of approximately three years’ duration. Her symptoms initially consisted of mild exertional dyspnea and a dry cough. She sought medical attention within approximately 1 week of symptom onset and received symptomatic treatment, including antihistamines, with partial improvement. Over the following years, similar episodes recurred and were managed intermittently with symptomatic medications. Her dyspnea gradually progressed to marked exertional limitation; she remained independently ambulatory but developed tachypnea while climbing stairs and had to stop to rest. The cough later became productive of small amounts of white sputum, which was occasionally streaked with scant blood. She also reported right-sided chest pain but denied fever, night sweats, weight loss, or prior recurrent respiratory infections. There was no history of thoracic surgery, chronic pulmonary disease, or known parasitic infection. She lived in a rural livestock-raising area and had regular contact with sheep and dogs.

Following persistent symptoms, she underwent chest radiography at a local hospital, where a large right-sided thoracic opacity was identified, and CT was recommended. She subsequently presented to our emergency department and was admitted to the thoracic surgery service. Surgery was performed on the day following admission.

On admission, she was alert and hemodynamically stable. Her blood pressure was 120/70 mmHg, heart rate 80 beats per minute, respiratory rate 24 breaths per minute, temperature 37°C, and peripheral oxygen saturation of 95% on room air. She weighed approximately 50 kg and was 152 cm tall, corresponding to a body mass index of approximately 21.6 kg/m^2^. She did not require supplemental oxygen before surgery. General examination revealed mild pallor without peripheral edema, digital clubbing, or lymphadenopathy. Chest examination showed mild leftward tracheal deviation, markedly reduced right-sided chest expansion, absent tactile vocal fremitus, dullness to percussion, and almost completely absent breath sounds over the right hemithorax. Cardiac, abdominal, neurologic, and musculoskeletal examinations were otherwise unremarkable.

Laboratory investigations showed a white blood cell count of 7600/µL, with 77% neutrophils and 2% eosinophils, hemoglobin of 11.1 g/dL, hematocrit of 34%, and a platelet count of 500 000/µL. Serum urea was 34 mg/dL, and creatinine was 0.5 mg/dL. Alanine aminotransferase and aspartate aminotransferase were 16 U/L and 8 U/L, respectively. Prothrombin activity was 92%, and the international normalized ratio was approximately 1.0. Preoperative inflammatory markers, partial thromboplastin time, and arterial blood gases were not obtained. Pulmonary function testing was not performed because of concern that forced respiratory maneuvers might precipitate rupture of the giant tense cyst.

Serologic testing using an *E. granulosus* antibody assay was positive. Electrocardiography showed a regular sinus rhythm without significant abnormalities. Preoperative echocardiography was not performed because of the patient’s young age, absence of cardiac symptoms or history, and the planned early operative intervention.

**Figure 1.**
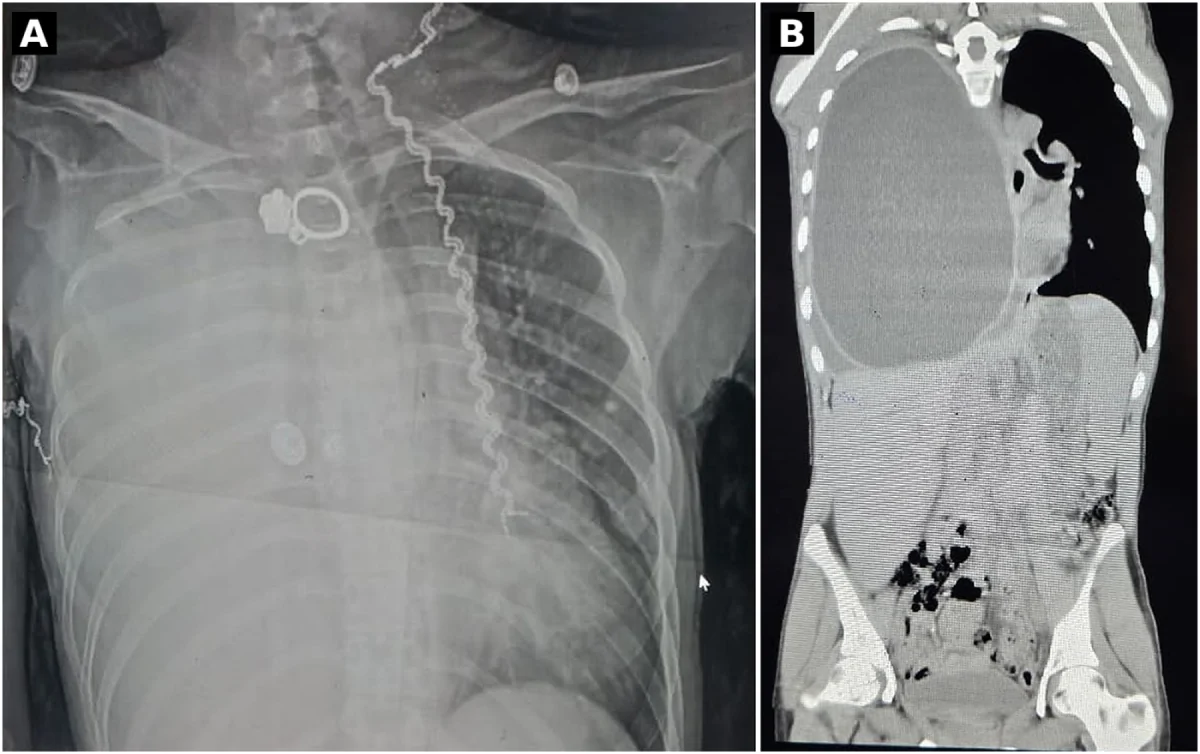
**Preoperative radiological findings. (A)** Frontal chest radiograph showing near-complete opacification of the right hemithorax with marked leftward displacement of the mediastinum and compression of the right lung. **(B)** Coronal contrast-enhanced computed tomography image demonstrating a single, well-defined, fluid-filled giant cyst occupying almost the entire right hemithorax, with severe compression of the residual right lung and substantial displacement of the mediastinum and heart to the left.

Chest radiography demonstrated a large homogeneous opacity occupying almost the entire right hemithorax, with marked leftward displacement of the mediastinum (Fig. 1A). Contrast-enhanced CT of the chest, abdomen, and pelvis showed a single, well-defined, fluid-filled cystic lesion measuring approximately 40 × 25 × 25 cm and occupying almost the entire right hemithorax. The lesion caused severe compression of the right lung and substantial leftward displacement of the mediastinum and heart (Fig. 1B). No additional cysts were identified in the contralateral lung, liver, abdomen, or pelvis. The diagnosis of a giant pulmonary hydatid cyst was supported by the epidemiologic exposure, characteristic imaging findings, positive serology, and subsequent intraoperative appearance. Histopathological confirmation was not available because the removed membrane was not submitted for pathological examination.

The patient was prepared for surgical excision. Preoperative albendazole was not administered because the treating team was concerned that altering the membrane of this extremely large, tense pulmonary cyst might increase its susceptibility to rupture. She received hydrocortisone 200 mg intravenously and cefazolin 1 g intravenously before the induction of anesthesia. A prophylactic antihistamine was not administered, although epinephrine was prepared for the management of a possible hypersensitivity reaction.

General anesthesia was induced, and a 35-Fr left-sided double-lumen endotracheal tube was inserted. Placement required three attempts because of distorted thoracic anatomy, marked leftward mediastinal displacement, and a relatively high laryngeal position. Correct positioning was confirmed by flexible bronchoscopy and auscultation. No intraoperative oxygen desaturation or documented increase in airway pressure occurred. One-lung ventilation lasted approximately 30 minutes. Detailed intraoperative ventilator settings and arterial blood gas values could not be retrieved from the available records.

**Figure 2.**
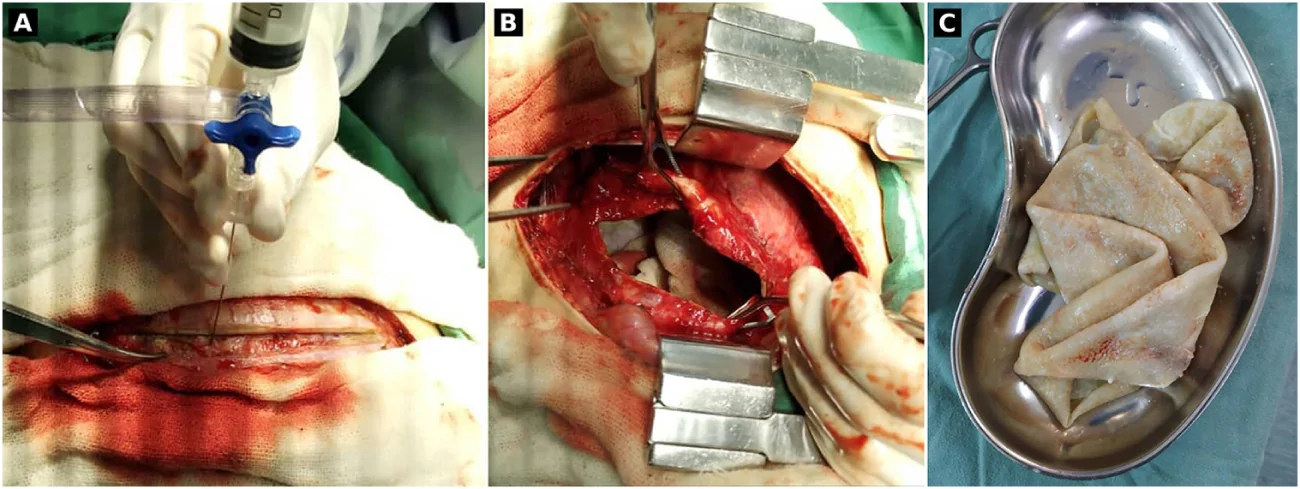
**Intraoperative findings during surgical management of the giant pulmonary hydatid cyst. (A)** Controlled needle aspiration of the tense cyst before full rib retraction to reduce intracystic pressure and minimize the risk of pleural spillage. Approximately 4 L of clear cyst fluid were aspirated. **(B)** Operative view through the right posterolateral thoracotomy after decompression and opening of the pericyst, showing the residual cystic cavity and the markedly compressed underlying lung parenchyma. **(C)** Macroscopic appearance of the extracted germinal membrane (endocyst), removed almost completely in one piece and displayed in a kidney dish as a large, folded, whitish membrane.

The patient was placed in the left lateral decubitus position, and a right posterolateral thoracotomy was performed through the sixth intercostal space. A tense, smooth-walled giant cyst occupied almost the entire right hemithorax and markedly compressed the residual lung. Because of the severe intracystic tension, controlled needle aspiration was performed before full rib retraction. Approximately 4 L of clear, watery fluid was aspirated over 5 minutes (Fig. 2A). No visible spillage into the pleural cavity occurred. Gauze packs soaked in hypertonic saline were placed around the operative field to minimize contamination.

After decompression, 500 mL of 20% hypertonic saline was instilled into the cyst cavity and allowed to remain for approximately 10 minutes before aspiration. The pericyst was then opened, exposing the residual cystic cavity and the severely compressed underlying lung parenchyma (Fig. 2B). The germinal membrane was dissected and almost completely removed in one piece. It appeared as a large, folded, whitish membrane without gross pus, foul odor, or macroscopic evidence of secondary infection (Fig. 2C). Neither the cyst fluid nor the membrane was submitted for microbiological culture.

Approximately five bronchial openings were identified in the residual cavity and closed individually with figure-of-eight 2-0 polyglactin sutures. An air-leak test was performed and demonstrated only a mild residual alveolar leak. Capitonnage was then completed using 0 polyglactin sutures. The residual lung parenchyma appeared viable. Gradual re-expansion and a recruitment maneuver were performed; however, complete re-expansion was not achieved. The right upper lobe, which had been most severely compressed by the cyst, failed to expand adequately, while expansion of the middle and lower lobes was partial but acceptable. A 28-Fr apical pleural chest tube was inserted and connected to low-pressure suction. Estimated intraoperative blood loss was approximately 300 mL. The operation lasted approximately 2.5 hours, in addition to approximately 30 minutes of anesthetic preparation.

Immediately after evacuation of approximately 4 L of cyst fluid, the patient developed progressive hypotension with tachycardia. Her blood pressure decreased from approximately 110/70 mmHg to a nadir of approximately 60/40 mmHg. There was no associated urticaria, flushing, facial edema, bronchospasm, wheezing, increased airway pressure, or intraoperative hypoxemia. Because of the close temporal relationship between cyst evacuation and hemodynamic collapse, a possible anaphylactic or anaphylactoid reaction was suspected and treated empirically. She received 0.5 mL of 1:1000 epinephrine intramuscularly on four occasions. Because hypotension persisted, continuous intravenous norepinephrine was initiated at approximately 0.128 µg/kg/min, resulting in a temporary improvement in blood pressure to approximately 100/50 mmHg.

Pneumomediastinum was also observed intraoperatively, with visible inflation of the mediastinal tissues during positive-pressure ventilation. The mediastinum was explored, but no major macroscopic tracheal or bronchial injury was identified. A small occult airway injury related to the difficult intubation attempts or barotrauma was considered possible, although the source of the air leak was not definitively established. No subsequent diagnostic bronchoscopy or postoperative CT was performed.

The patient remained hemodynamically unstable and was transferred directly to the intensive care unit while intubated and receiving norepinephrine. Extubation was not attempted because of persistent circulatory instability, although oxygenation was initially acceptable. Approximately 2 hours after surgery, she received two units of whole blood. No plasma or platelet transfusions were administered. The exact volumes of intraoperative crystalloids were unavailable, although overall recorded fluid input and output were approximately balanced.

**Figure 3.**
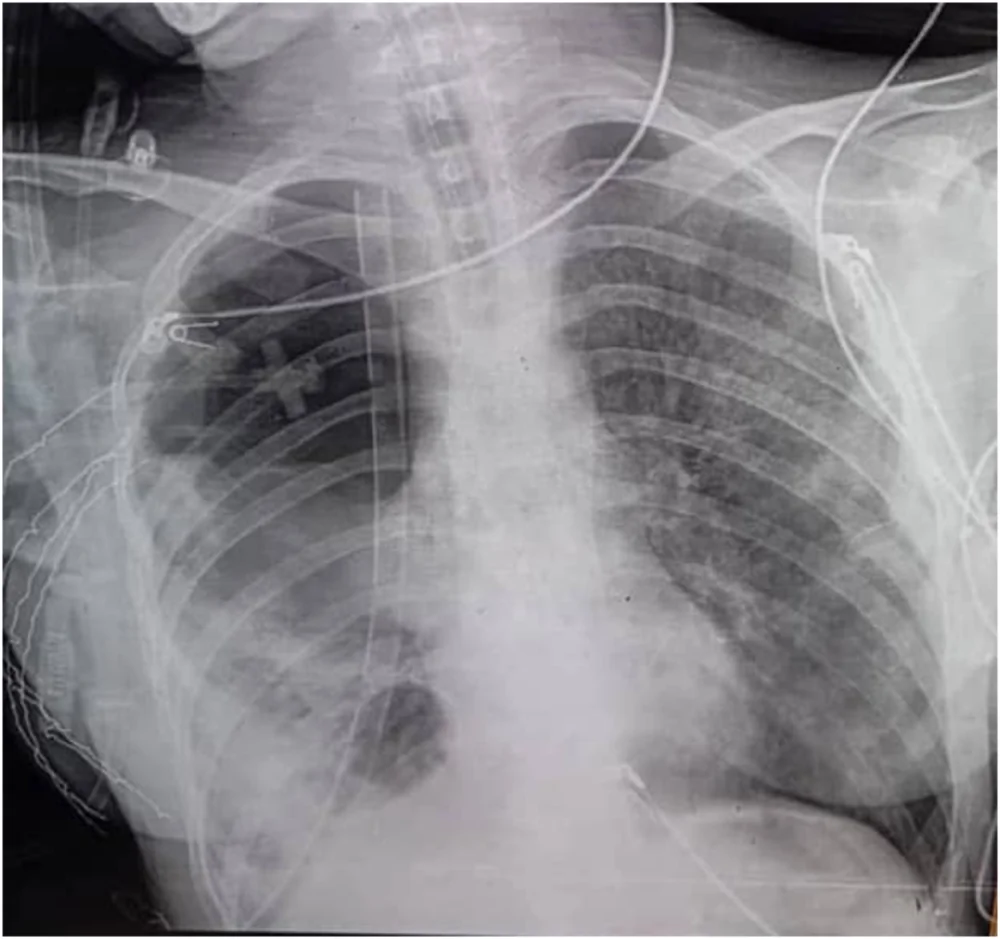
Portable anteroposterior chest radiograph obtained approximately 3 hours after surgery shows a right pleural chest tube, persistent right pleural air with incomplete re-expansion of the right lung, and bilateral diffuse alveolar–interstitial pulmonary opacities representing early postoperative lung injury.

A portable chest radiograph, obtained approximately 3 hours after surgery, showed a right pleural chest tube, persistent right pleural air with incomplete re-expansion of the right lung, and bilateral diffuse alveolar–interstitial pulmonary opacities (Fig. 3). During the subsequent hours, her respiratory status deteriorated, and within approximately 6 hours, she developed severe hypoxemic respiratory failure with progressive bilateral pulmonary opacities consistent with acute respiratory distress syndrome. Progressive metabolic acidosis became evident approximately 18 hours after surgery. Detailed ventilator settings, serial arterial blood gas values, and complete hemodynamic trends could not be retrieved from the available records.

Postoperative transthoracic echocardiography showed normal biventricular systolic function, normal estimated pulmonary pressures, no significant valvular abnormality, and no pericardial effusion, making primary cardiac pump failure an unlikely explanation for the shock. Management in the intensive care unit included vasopressor support, lung-protective mechanical ventilation, recruitment maneuvers, hydrocortisone 100 mg twice daily, inhaled nitric oxide, and broad-spectrum antimicrobial therapy. Extracorporeal membrane oxygenation was not available.

Despite maximal available supportive treatment, the patient developed refractory circulatory failure, worsening hypoxemia, metabolic acidosis, and progressive multiorgan dysfunction. The intensive care team considered septic shock to be the terminal mechanism; however, no definitive single cause of the deterioration could be established. The overall course was considered likely multifactorial, with possible contributions from an immediate hypersensitivity-type reaction following cyst evacuation, systemic inflammatory response, rapid re-expansion of the chronically compressed lung, perioperative lung injury, and possible occult airway trauma. Cardiac arrest occurred on the third postoperative day, and resuscitation was unsuccessful. The fatal postoperative outcome was classified as Clavien–Dindo grade V. The chronological clinical course is summarized in Table 1.

**Table 1:** Structured timeline of the clinical course.

Time interval	Clinical stage	Key events and management
Approximately 3 years before admission	Initial symptoms	Mild exertional dyspnea and dry cough began.
Within approximately 1 week of symptom onset	First medical assessment	Symptomatic treatment, including antihistamines, produced partial temporary improvement.
During the following years	Progressive clinical course	Recurrent symptoms gradually progressed to marked exertional limitation, productive cough with occasional scant blood-streaked sputum, and right-sided chest pain.
Day before surgery	Hospital admission and diagnosis	Chest radiography and computed tomography demonstrated a solitary giant right pulmonary cyst with severe lung compression and marked mediastinal displacement. Serology for *Echinococcus granulosus* was positive.
Day 0	Surgical treatment	Right posterolateral thoracotomy was performed. Approximately 4 L of clear cyst fluid were aspirated, the endocyst was removed, bronchial openings were closed, and capitonnage was completed.
Immediately after cyst decompression	Hemodynamic collapse	Progressive hypotension and tachycardia developed. A suspected anaphylactic or anaphylactoid reaction was treated with intramuscular epinephrine and continuous norepinephrine.
Immediately postoperatively	Transfer to intensive care	The patient remained intubated and was transferred directly to the intensive care unit because of persistent hemodynamic instability; oxygenation was initially acceptable.
Approximately 2 hours postoperatively	Blood transfusion	Two units of whole blood were administered.
Approximately 3 hours postoperatively	Early pulmonary deterioration	Chest radiography showed incomplete right lung re-expansion, persistent pleural air, and bilateral diffuse pulmonary infiltrates.
Within approximately 6 hours postoperatively	Severe respiratory failure	Severe hypoxemia developed with a clinical-radiographic picture consistent with acute respiratory distress syndrome.
Approximately 18 hours postoperatively	Metabolic deterioration	Progressive metabolic acidosis became evident.
Postoperative days 1–3	Refractory critical illness	Persistent circulatory failure, worsening hypoxemia, and progressive multiorgan dysfunction continued despite vasopressor, ventilatory, corticosteroid, inhaled nitric oxide, and antimicrobial support.
Postoperative day 3	Outcome	Cardiac arrest occurred, and resuscitation was unsuccessful. The outcome was classified as Clavien-Dindo grade V.

All intervals are reported relative to symptom onset, surgery, or the postoperative period.

## Discussion

Pulmonary hydatid cysts may reach exceptional dimensions in children and young adults because the compliance of the lung and thoracic cage permits gradual expansion before severe functional impairment develops^[^7,9–11^]^. In this patient, the prolonged clinical course likely allowed progressive lung compression and major mediastinal displacement. The diagnosis was strongly supported by exposure in an endemic area, characteristic imaging, positive *E. granulosus* serology, and typical intraoperative endocyst morphology. However, pulmonary serology is supportive rather than definitive, and the absence of histopathological confirmation remains an important diagnostic limitation^[^3–5,12,13^]^.

Surgery was justified by the severe mass effect, progressive symptoms, and bronchial communications. The objectives of pulmonary hydatid surgery are complete parasite removal, prevention of dissemination, closure of bronchial openings, and maximal preservation of viable lung parenchyma^[^6,11,13,14^]^. A recent analysis of 872 patients showed that cystotomy with capitonnage is feasible in most pulmonary cases and is associated with low recurrence and postoperative mortality^[^11^]^. In the present case, PAIR would have carried considerable risks of bronchial leakage, pleural contamination, secondary implantation, and hypersensitivity, while medical therapy alone could not promptly relieve the advanced mechanical compression.

Albendazole is commonly incorporated into pulmonary hydatid management, but its timing depends on cyst characteristics and clinical circumstances^[^6,11,15^]^. The decision to withhold preoperative therapy was consistent with concerns that albendazole may weaken the wall of an intact pulmonary cyst and increase its susceptibility to rupture, whereas postoperative administration is used to reduce recurrence^[^11^]^. This was therefore a case-specific decision rather than evidence that preoperative albendazole is universally contraindicated.

The immediate circulatory collapse following cyst decompression raised concern for an anaphylactic or anaphylactoid reaction caused by antigen exposure, even in the absence of gross spillage. Hydatid rupture or antigen release may cause urticaria, bronchospasm, eosinophilia, or cardiovascular collapse^[^14,16–18^]^. However, the lack of cutaneous manifestations, bronchospasm, increased airway pressure, and intraoperative hypoxemia prevented definitive confirmation of classic anaphylaxis. Empirical epinephrine treatment was nevertheless reasonable because severe cardiovascular involvement may predominate.

Alternative causes of shock were less strongly supported. Hemorrhagic shock was unlikely because blood loss was limited and no vascular injury or concealed bleeding was identified. Primary cardiogenic shock was also improbable, given the preserved postoperative biventricular function and absence of significant valvular or pericardial abnormalities. Abrupt decompression of a chronically compressed hemithorax may nevertheless have altered venous return and pulmonary vascular flow, triggering inflammatory mediator release. These mechanisms could have acted concurrently rather than being mutually exclusive diagnoses.

The intraoperative pneumomediastinum suggests an additional potential mechanism of injury. Although no major tracheobronchial disruption was identified, a small airway microinjury related to repeated placement of a double-lumen tube could not be excluded. Barotrauma during positive-pressure ventilation was also a possibility. Because postoperative bronchoscopy and CT were unavailable, the origin and clinical significance of the mediastinal air remained uncertain.

Re-expansion pulmonary injury was an important consideration in the early postoperative deterioration. Chronic lung compression, rapid large-volume decompression, and attempted re-expansion are recognized risk factors for increased pulmonary vascular permeability, ischemia–reperfusion injury, and pulmonary edema^[^19^]^. Re-expansion injury commonly develops within the first few hours after drainage or lung re-expansion and may range from limited radiographic abnormalities to severe respiratory and circulatory failure^[^19^]^. In this case, incomplete and heterogeneous re-expansion may also have exposed the residual lung to uneven mechanical stress.

The rapid development of severe hypoxemia with bilateral pulmonary opacities, in the absence of echocardiographic evidence of cardiac failure or documented, marked fluid overload, was clinically and radiographically consistent with acute respiratory distress syndrome (ARDS)^[^20^]^. Shock, transfusion, aspiration, and systemic inflammation are recognized precipitating factors, and more than one may coexist^[^20^]^. Nevertheless, formal retrospective classification of ARDS severity was not possible because serial PaO_2_/FiO_2_ values, complete ventilator settings, and PEEP measurements were unavailable.

Other potential contributors were considered but could not independently explain the course. A major aspiration of cyst contents was less likely because no gross spillage was observed. Transfusion-related acute lung injury could not be excluded because the first documented bilateral pulmonary abnormalities occurred after transfusion; however, the concurrent shock, cyst decompression, and attempted lung re-expansion prevented attribution to a single mechanism.Sepsis was considered by the intensive care team and treated empirically; however, the absence of cultures, serial inflammatory markers, and complete hemodynamic data prevents confirmation of infection as the sole terminal mechanism.

The fatal deterioration was therefore most plausibly multifactorial, involving an initial hypersensitivity or hemodynamic insult followed by systemic inflammatory activation, re-expansion lung injury, severe ARDS, and possible occult airway trauma, with superimposed infection remaining possible. The outcome does not indicate that surgery was inappropriate because the profound mass effect and bronchial communications made observation, PAIR, and medical treatment alone unsuitable. Rather, it demonstrates that anatomical success does not guarantee physiological recovery after decompression of a chronically compressed hemithorax.

## Conclusion

Giant pulmonary hydatid cysts can produce severe physiological compromise despite technically successful surgical excision. In this case, rapid postoperative deterioration, with refractory shock and ARDS, was likely multifactorial, with possible contributions from hypersensitivity, systemic inflammation, and re-expansion lung injury. These lesions require careful multidisciplinary planning, controlled decompression, gradual lung re-expansion, and immediate access to advanced perioperative critical care.

## Data Availability

Not applicable.
